# Finger millet and soybean as functional ingredients in next-generation fermented foods: a review of nutritional, technological, and health-promoting perspectives

**DOI:** 10.3389/fnut.2026.1718090

**Published:** 2026-03-31

**Authors:** Phindiswa Kokisi, Felix Nchu, Learnmore Kambizi, Callistus Bvenura

**Affiliations:** Department of Horticultural Sciences, Cape Peninsula University of Technology, Bellville, South Africa

**Keywords:** agroecology, sustainability, bioavailability, fermentation, finger millet, functional foods, microbial ecology, soybean

## Abstract

**Introduction:**

The ever-increasing global malnutrition, environmental degradation, and food insecurity challenges have intensified interest in sustainable food systems and underutilized crops. Fermentation improves the nutritional quality, digestibility, shelf life, and sensory attributes of plant-based foods. Finger millet (*Eleusine coracana*) and soybean (*Glycine max*) are promising for functional food development due to their complementary nutrient profiles. Finger millet is rich in minerals, fibre, and polyphenols, while soybean provides high-quality protein and bioactive compounds. Their synergistic amino acid profiles and the benefits of fermentation—such as improved micronutrient bioavailability and reduced antinutritional factors—make them suitable for developing innovative fermented foods. Therefore, this review evaluates the nutritional value, fermentation potential, and health-promoting properties of finger millet and soybean for sustainable nutrition and food security.

**Methods:**

A narrative review following PRISMA principles was conducted using Google Scholar, PubMed, ScienceDirect, and Scopus. Literature from 2000–2025 on finger millet, soybean, fermentation, and functional foods was searched, yielding 116 records. After screening, 59 peer-reviewed studies were included. Two reviewers independently extracted and analysed data through thematic synthesis on nutritional composition, fermentation methods, microbial ecology, functional properties, and health benefits.

**Results:**

The literature shows that fermentation significantly enhances the nutritional and functional value of both crops. Fermentation reduces antinutritional factors such as phytates and tannins, improves protein digestibility, and increases mineral bioavailability. Lactic and acetic acid fermentation also enhance flavour, texture, and shelf stability. However, the review identified a major research gap: few documented fermented foods combine finger millet and soybean despite their complementary nutritional profiles.

**Discussion:**

Finger millet and soybean present strong potential for developing next generation fermented functional foods that address malnutrition, lactose intolerance, and dietary protein deficiencies. Nevertheless, several challenges remain, including fermentation standardization, sensory acceptance, limited infrastructure, and insufficient characterization of microbial communities and bioactive metabolites. Advancing multi-omics research, improving fermentation technologies, and promoting supportive policies and value chains will be critical for translating these crops into scalable, sustainable food innovations.

## 1 Introduction

The global rise in malnutrition, environmental degradation, and the pressing need for sustainable food systems has shifted academic and industrial discourse toward the study of underutilized crops and innovative food processing technologies (1). These methods are proven to expand food variety, ensure proper nutrient intake, and support culturally appropriate diets, particularly in low-income communities (2, 3). Consequently, plant-based functional fermented foods stand out as a powerful solution, offering superior nutrition and substantial health benefits (3). Fermentation, an ancient yet dynamic biotechnological technique, plays a crucial role in improving the shelf-life, organoleptic characteristics, and overall nutritional quality of food products (4, 5). Globally, fermented foods constitute about 20%–40% of the human diet, a figure that continues to rise in response to the increasing consumer demand for functional and sustainable dietary options (2, 6). Importantly, the functional attributes of fermentation include the reduction of antinutritional compounds, enhancement of micronutrient bioavailability, and improved digestibility of plant-derived proteins (4, 5). Within the spectrum of promising plant-based ingredients for functional food innovation, finger millet and soybean have garnered significant scholarly attention due to their complementary nutrient compositions and documented health benefits. Finger millet delivers high levels of iron, calcium, dietary fiber, and polyphenols, directly supporting bone health, blood sugar control, and antioxidant defense (7). Soybean is a complete source of plant-based protein and is rich isoflavones, bioactive compounds linked to cholesterol reduction and enhanced metabolic function (8, 9). Critically, the amino acid profiles of these two crops are synergistic, i.e., finger millet is deficient in lysine that is one the building blocks protein required for the synthesis of enzymes, hormones, antibodies and structural proteins such as collagen and elastin, and soybean lacks adequate methionine that is a sulphur-containing amino acid, and it plays a critical physiological and biochemical roles and enables a balanced protein profile when combined (10). In addition to this, both crops are important plant-based protein sources that are complete in terms of nutrition and culture (10). Therefore, this review critically explores the current body of literature concerning the nutritional properties, functional applications, and health-related effects of finger millet and soybean in the context of plant-based fermented food development. Particular attention is directed toward the role of fermentation in improving the functional attributes of these crops and their combined potential to address prevalent dietary deficiencies, including lactose intolerance and protein-energy malnutrition (11). By synthesizing interdisciplinary evidence from nutritional science, food biotechnology, and public health domains, this study aims to elucidate the opportunities and challenges associated with leveraging finger millet and soybean in the creation of nutritionally enriched, culturally relevant fermented foods (12). It also identifies prevailing research trends, existing knowledge gaps, and future directions for innovation in the development of next-generation functional plant-based products (12).

## 2 Literature search and data collection

**Figure 1:**
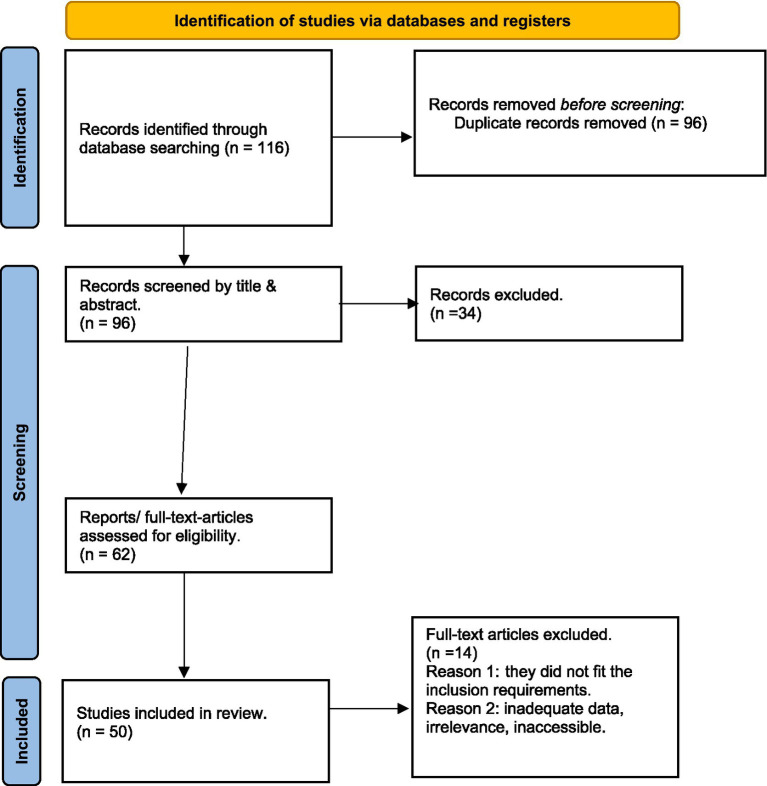
The diagram illustrates the complete literature search and data compilation process, detailing the methodology implemented in accordance with the PRISMA framework.

This review was conducted across major scientific databases such as Google scholar, PubMed, Science Direct, and Scopus following PRISMA (Preferred Reporting Items for Systematic Reviews and Meta-Analyses) guidelines. While this review was conducted in alignment with PRISMA principles to ensure transparency and rigor, it was designed as a narrative review rather than a strictly systematic review. The search strategy was implemented between May and November 2025 and included publications from 2000 to 2025 to capture contemporary developments in plant-based fermentation. Database specific search strings were adapted for each platform using combinations of the following keywords: “finger millet,” “soybean,” “fermentation of millets and legumes”, “functional foods”, “nutritional properties”, “technological applications”, and “health benefits.” The selection process proceeded as follows: the initial search resulted in 116 articles. After deleting 96 duplicates, 96 articles were screened using the title and abstract. Thirty-four publications were eliminated because they did not fit the inclusion requirements (for example, being unrelated to food science or not addressing fermentation or functional foods of both crops). A total of 62 full-text papers were evaluated, with 14 being eliminated due to inadequate data, lack of relevance, or lack of access to the full text. In addition, a further nine articles were added during the review process. Finally, this review comprises 59 high quality papers that are pertinent. Figure 1 simplifies this data by presenting a complete diagram of the entire selection process. Despite the extensive research into the individual nutritional and health-promising attributes of finger millet and soybean, as well as their technological potential in food processing, the review revealed a notable gap. At present, there are no commercially available or well-documented fermented foods that use these crops together or on their own. Consequently, data compilation primarily drew from studies on separate fermentation of related legumes and cereals, as well as the biochemical and functional properties of finger millet and soybean that suggest their promising suitability for future fermentation applications.

The PRISMA framework was applied systematically through four stages, viz.: identification, screening, eligibility, and inclusion. During the identification stage, multiple databases mentioned above were searched independently to minimize publication bias and ensure broad literature coverage. Screening was conducted using predefined inclusion and exclusion criteria to reduce selection bias as mentioned above. Only peer reviewed journal articles published in English were included. This language restriction was applied due to limitations in reliable translation and to ensure methodological consistency. Publication bias was further minimized by cross-checking reference lists of relevant reviews and primary studies, and by avoiding exclusive reliance on a single database. All search results were exported to a reference manager, where duplicates were removed prior to screening. Grey literature sources such as thesis, technical reports, conference proceedings, and non-peer reviewed documents were not included in this review. Preprints and unpublished manuscripts were also excluded to ensure that only rigorously peer reviewed and validated studies were analysed.

### 2.1 Screening and reviewer workflow

Literature screening and data extraction were performed independently by two reviewers. Titles and abstracts were first screened to determine potential relevance, followed by full text evaluation of eligible studies. Disagreements regarding inclusion or interpretation were resolved through discussion and consensus. Where consensus could not be reached, a third senior reviewer was consulted to make the final decision. Although formal statistical analysis of inter reviewer agreement was not conducted, the dual reviewer system and consensus ensured consistency and minimized subjective bias.

In addition, data from included studies were analysed using a structured thematic synthesis approach. Extracted information was coded into predefined categories including nutritional compositions, fermentation techniques, microbial strains, functional properties, ad health benefits. Studies were further sub-grouped based on crop type, product category, and fermentation method. The final synthesis was organized to present evidence for each crop individually followed by integrated analysis.

A meta-analysis was not performed due to substantial heterogeneity among available studies. The reviewed literature differed markedly in experimental design, fermentation substrates, microbial strains, analytical techniques, and outcome measures. For example, some studies assessed nutritional enhancements using *in vitro* mineral bioavailability assays, while others relied on animal models or sensory evaluation. These inconsistencies pretend meaningful statistical pooling of data, making a narrative synthesis more appropriate.

### 2.2 Review framework and scope

This review was guided by an adapted PICOS (Population Intervention Comparator Outcomes Study design) framework to ensure conceptual clarity and alignment of included evidence. The population of interest comprised studies involving finger millet, soybean, or composite fermented plant-based foods. The interventions included natural, spontaneous; starter culture mediated fermentation processes. No formal comparator was required, as the review synthesized diverse study designs. Primary outcomes included changes in nutritional composition, bioavailability, functional properties, and microbial ecology. Eligible study designs included experimental, observational, and analytical studies published in peer reviewed journals. Adoption of this framework provided a structured basis for literature selection and thematic synthesis.

In addition, in this review, a deliberate distinction is made between empirical scientific evidence and ethnobotanical or traditional knowledge. Traditional practices are discussed to provide cultural and historical context, while conclusions regarding nutritional and functional benefits are based exclusively on peer reviewed experimental findings.

### 2.3 Millets

Millets are among the earliest cultivated grain crops in human history (13). These cereals are broadly categorized into two main groups called pearl and minor millets. Pearl millets include *Pennisetum glaucum*, *P. typhoides, P. tyhpideum*, and *P. americanum*, while minor millets encompass species such as finger millet (*Eleusine coracana*), proso millet (*Penicum miliaceum*), foxtail millet (*Setaria italica*), barnyard millet (*Echinochloa crugalli* and *Echinochloa colona*), little millet (*Panicum sumatrense*), and kodo (*Paspalum scrobiculatum*) (13). Furthermore, minor millets are defined by their notably smaller seed size, setting them apart within the millet family (13). The latter group encompasses species such as finger millet (*Eleusine coracana*), proso millet (*Penicum miliaceum*), foxtail millet (*Setaria italica*), (*Echinochloa crugalli* and *Echinochloa colona*, *Panicum sumatrense*), and (*Paspalum scrobiculatum*). Table 1, distinguishes finger millet from other millets and their region of origin (14). While the precise origins of these crops remain debated, compelling evidence indicates that finger millet was independently domesticated in several regions, with East and West Africa, alongside East and South Asia, leading this advancement (13). For instance, in parts of Asia, millets are believed to have been cultivated even before rice. Notably, proso and foxtail millets were domesticated in China nearly 10,000 years ago, underscoring their long-standing importance in both agriculture and culture (13). Similarly, kodo and little millets were domesticated in South Asia between 3,700 and 5,000 years ago. However, pearl millet traces its domestication to West Africa, particularly the Sahel region, which historical evidence suggestion its cultivation as early as 8,000 years ago (13). Moreover, finger millet is native to the highlands of East Africa, particularly Ethiopia and Uganda, where it was first domesticated around 5,000 years ago. Nonetheless, millets remain underutilized globally, even though they have a long history and excel in dry and semi-arid climates (13). Although millets are the sixth most-produced cereal crop, their impact on global food systems remains minimal despite their production scale. Bvenura and Kambizi (13), stated that during the 2019/2020 growing season, major producers included India, Niger, China, and Nigeria, collectively yielding a substantial portion of the estimated 27.8 million tonnes of global millet production. Moreover, Africa leads global millet consumption, representing over 40% of the total demand (13). Additionally, millets are recognized for their resilience to climate variability and their vital role in strengthening food security, particularly in areas with unpredictable rainfall and poor soil fertility (14). Their nutritional richness and suitability for marginal environments position them as vital candidates for future sustainable agriculture (14).

**Table 1:** Distinguishing finger millet from other millets.

Feature	Finger millet (*Eleusine coracana*)	Pearl millet (*Pennisetum glaucum*)	Proso millet (*Panicum miliaceum*)	Foxtail millet (*Setaria italica*)
Origin	Ethiopian and Ugandan highlands (East Africa).	Sahel region of Africa.	Central Asia.	China and parts of Europe.
Climate preference	Cooler, higher altitudes, moderate rainfall (500 mm).	Hot, dry, low rainfall, thrives in hottest, driest areas	Temperate to subtropical dry regions	Temperate regions, mainly Asia and Europe.
Growth altitude	Up to 2,400 m above sea level.	Generally low altitude, hot climates.	Low to moderate altitudes.	Low to moderate altitudes.
Grain use	Food staple, traditional beer, high nutritional value.	Food staple, fodder.	Bird seed, food in parts of Asia.	Food, fodder.
Drought tolerance	High, but prefers moderate rainfall.	Very high, adapted to extreme drought.	Moderate	Moderate
Cultivation area	Africa (Ethiopia, Uganda, Kenya), South Asia (India, Nepal).	Africa and South Asia.	Asia, North America (bird seed).	Asia (China), Europe.

Bvenura and Kambizi (13); Kumar et al. (14).

#### 2.3.1 Finger millet

**Figure 2:**
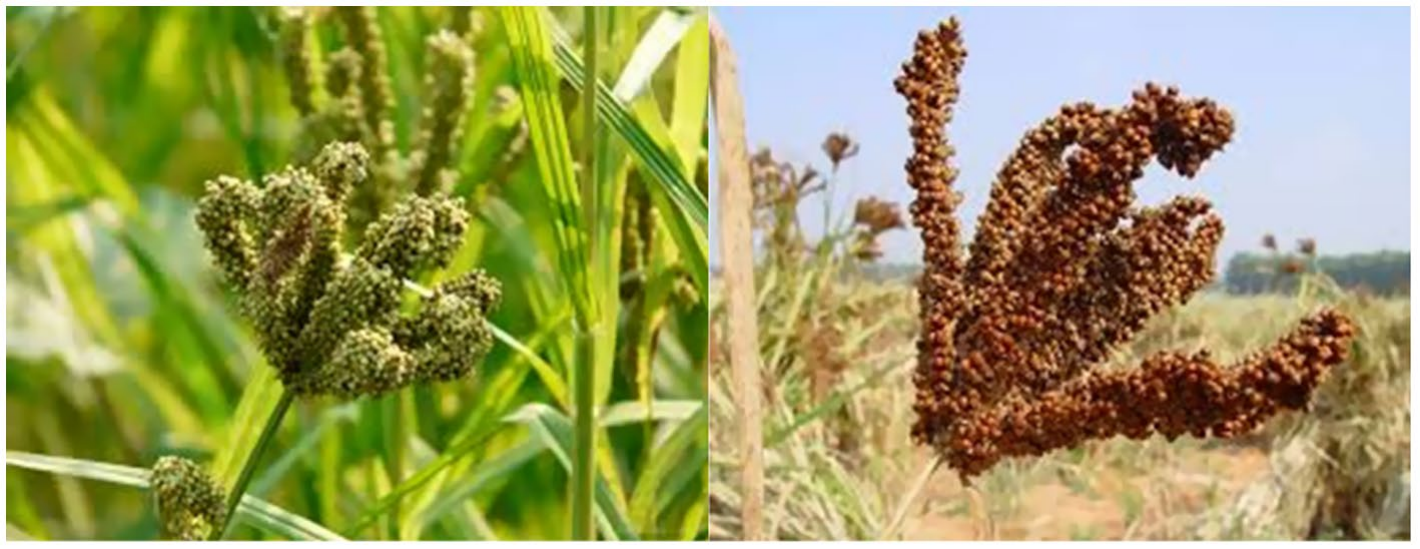
Colour transition of finger millet grains from green to yellowish-brown at maturity.

Specifically, *Eleusine coracana* (finger millet) is a small-seeded cereal traditionally cultivated across Africa and parts of Asia, as shown in Figure 2, at the ripening stage in the field undergoes a transition in grain-color from green to a yellowish-brown (14). Finger millet is drought-tolerant, nutrient-rich, and thrives under low-input conditions, making it an ideal crop for sustainable agriculture in dryland regions (15). Its high water uses efficiency, adaptive morphological traits, and robust stress-response mechanisms position it as a key resource for resilient food systems in challenging environments (15). It is an annual, self-pollinating tetraploid specie, notable for its resilience to drought, adaptability to low-fertility soils, and resistance to pests and diseases, including during storage (15). Moreover, finger millet flourishes at elevations up to 2,400 m and performs well in cooler conditions, offering a distinct advantage over pearl millet and sorghum in highland areas (14).

#### 2.3.2 Origin and distribution of finger millet

According to Singh (60), finger millet originated about 5,000 years ago in the highlands of East Africa, primarily Ethiopia and Uganda, through the domestication of its wild progenitor, *Eleusine coracana* subsp. *africana*. Furthermore, finger millet reached the Indian subcontinent around 3,000 years ago, where it became integral to regional agriculture and established a secondary hub of genetic diversity (60). Today, its cultivation is concentrated in Africa and South Asia, with leading producers such as India, Ethiopia, Nepal, Uganda, Kenya, and Tanzania. It is also grown in southern Sudan and extends as far south as Mozambique (60).

#### 2.3.3 Taxonomic and botanical description of finger millet

*Eleusine coracana* is part of the Poaceae family, a group that includes the world’s key cereal crops (16). It is an allotetraploid species with chromosome number 2n = 4x = 36 (16). The cop’s adaptability to diverse environments is attributed to its genetic makeup and evolutionary history (16). *Eleusine coracana* belong to the kingdom Plantae, subkingdom Tracheobionta (vascular plants), superdivision Spermatophyta (seed plants), division Magnoliophyta (angiosperms), class Liliopsida (monocots), subclass Commelinidae, order Poaceae (grass family), subfamily Chloridoideae, tribe Eragrostidae, and genus *Eleusine* (16).

#### 2.3.4 Secondary metabolites in finger millet

Finger millet serves as an essential source of nutrition for low-income communities, especially in developing countries (15). However, despite its considerable nutritional richness, it remains largely underutilized and is often overshadowed by more commonly consumed grains (15). The grain’s natural brown color and gluten-free quality make it ideal for those with gluten sensitivities (15). Nutritionally, finger millet stands out for its high levels of carbohydrates, dietary fiber, key minerals, and a wide range of both essential and non-essential amino acids (such as valine, methionine, tryptophan, and leucine) and non-essential (including alanine, glutamic acid, and aspartic acid) (2). These amino acids are critical for cellular function and overall human development. Moreover, finger millet contains a diverse array of bioactive compounds, including tannins, flavonoids, polyphenols, and steroids shown in Table 2, which significantly contribute to its functional health benefits (2). According to Seiphitlhile et al. (15), the consumption of finger millet has been shown to aid in the prevention and management of non-communicable diseases such as diabetes, hypertension, and gastrointestinal disorders. Its high phytochemical content drives finger millet’s antioxidant, anti-aging, metabolic, and cardiovascular benefits (15). Therefore, analysing its phytochemical profile is essential to fully understand the grain’s health-promoting properties beyond simple nutrition (15). Furthermore, this research is essential for formulating evidence-based strategies that promote finger millet as a functional food, directly addressing the rise of non-communicable diseases (15). By highlighting the pharmacological relevance of its phytochemicals, this research can facilitate the integration of finger millet into mainstream health-promoting diets and nutraceutical applications, as a result, this approach significantly enhances public health outcomes while reinforcing agricultural sustainability in resource-limited regions (15). Table 3 presents a detailed overview of secondary metabolites in finger millet.

**Table 2:** Biological activities and health functions of phytochemicals in finger millet.

Phytochemical compound	Biological function/health benefit
Polyphenols	Possess potent antioxidant properties that help neutralize free radicals, thereby reducing cellular oxidative damage. This activity is linked to the prevention of chronic diseases such as cancer, diabetes, and cardiovascular disorders (2, 15).
Flavonoids	Exhibit strong anti-inflammatory, antioxidant, and anti-aging effects. They may help modulate immune responses, improve vascular function, and protect against neurodegenerative diseases disorders (2, 15).
Tannins	In controlled amounts, tannins contribute to antimicrobial and anti-parasitic activities and can support gut health. However, excessive intake may interfere with nutrient absorption, particularly iron and protein digestibility disorders (2, 15).
Phytosterols	Structurally like cholesterol, phytosterols compete with dietary cholesterol for absorption in the digestive tract, thereby lowering LDL (bad) cholesterol levels and promoting heart health. They may also possess anti-cancer and anti-inflammatory properties (2, 15).

**Table 3:** Secondary metabolites in finger millet.

Secondary metabolite class	Specific compounds/metabolites	Reported range/levels	Biological/functional role
Phenolic acids	Ferulic acid, p-coumaric acid, gallic acid.	Significant variation among varieties, e.g., total phenolics (3.5–10.2 mg/g TAE).	Potent antioxidants; reduce oxidative stress (7).
Flavonoids	Quercetin, catechin, apigenin, & luteolin.	Total flavonoids (4.5–5.5 mg/g CE).	Anti-inflammatory, anti-aging, antimicrobial (7, 55).
Tannins	Condensed tannins, catechin derivatives	Levels vary with grain color which is brown and white varieties.	Antimicrobial, antioxidant (55).
Anthocyanins	Cyanidin derivatives	Increased during germination, variable by genotype.	Antioxidants; contribute to color and health benefits (55).
Alkaloids	Various unidentified alkaloids	Detected qualitatively in extracts.	Potential antimicrobial and defensive roles (7).
Terpenoids/sterols	Phytosterols and other terpenoids	Identified in extracts but quantitatively limited data.	Cardiovascular support, antioxidant properties (7).
Organic acids	Citric acid, lactic acid, itaconic acid	Varies with processing, fermentation increases levels.	Involved in metabolism and flavor, antimicrobial (55).

#### 2.3.5 Nutritional constituents of finger millet

Finger millet is a resilient cereal crop uniquely suited to semi-arid and subtropical climates, where it thrives under challenging conditions (7). Its impressive nutritional profile positions it as a key contributor to food and nutritional security in regions with limited agricultural options. However, it is high calcium, iron, and dietary fiber content, low glycemic index, and natural gluten-free quality (7). Because of its unique nutritional properties, finger millet is particularly beneficial for people with diabetes or gluten intolerance, including those with celiac disease (7). Moreover, it provides a balanced combination of carbohydrates, proteins, and fats. Table 4 details the key nutrients in finger millet and demonstrates their significant health advantages (17). Notably, finger millet is exceptionally rich in calcium and iron, nutrients essential for bone strength and preventing anemia, positioning this grain as a powerful tool for improving nutritional outcomes (17).

**Table 4:** Nutritional constituents of finger millet and their health functions.

Nutrient category	Constituent	Nutritional/health role	Examples
Proximate constituents	Carbohydrates (60–75%)Protein (6–13%)Fat (1–2%)Total fibre (11–18%)	Provides energy; contains resistant starch beneficial for diabetic diets.	Used in diabetic-friendly porridge or flatbreads to reduce glucose spikes (17).
		Contains essential amino acids (e.g., methionine); supports growth and repair.	Combined with legumes for complete protein in weaning foods or adult meals (7).
		Mainly unsaturated fatty acids (oleic and linoleic acids); supports cardiovascular health.	Used in heart-healthy snacks and low-fat diet formulations (7, 17).
		Promotes gut health; reduces cholesterol; improves satiety and weight management.	Used in high-fibre biscuits and wholegrain breakfast cereals (17).
Mineral constitutes	Calcium (280–344 mg/100 g).Iron (3.3–14.8 mg/100 g).Phosphorus (130–293 mg/100 g).Magnesium (120–140 mg/100 g)Zinc (0.4–3.2 mg/100 g).	Essential for bone health, especially in children, pregnant women, and elderly.	Added to infant porridge and bone-health supplements (56).
		Vital for hemoglobin formation and oxygen transport.	Beneficial in anemia-prevention diets for adolescent girls and pregnant women (56).
		Supports metabolism, immunity, and enzyme function.	Incorporated in school feeding and functional health products (56).
		It is important for enzyme function, muscle, and nerve functions.	Added to dietary supplements and health foods (56).
		Important for immune function and enzyme activity.	Included in fortified foods and school nutritional programs (56).

#### 2.3.6 Value added products from finger millet fermented

Finger millet can effectively substitute for rice and other starchy grains in various applications, offering versatility and nutritional benefits (61). Nevertheless, this approach is widely applied across various process technologies, including milling, malting, baking, popping, fermenting, roasting, and integrated processing methods, all of which play a crucial role in innovative product development (61). These strategies improve the consumption and value of finger millet (61). The most common finger millet fermented foods are fermented beverage, bread, pancakes/flatbreads, finger millet yogurt (dairy-free), snacks (crackers, crisps), finger millet-based fermented composite flour etc. (Table 5).

**Table 5:** Value-added products derived from fermented finger millet.

Product type	Description	Examples	Benefits
Fermented porridge	A traditional semi-liquid food prepared by fermenting finger millet flour in water.	Koozh (India), Obushera (Uganda)	Easily digestible, enhanced mineral bioavailability (iron, calcium), low glycemic index (2).
Fermented beverage	Non-alcoholic drink made by fermenting finger millet with water and sometimes natural sweeteners or spices.	Ambali, Finger millet probiotic drink	Probiotic, refreshing, promotes gut health (15).
Fermented bread	Bread made by incorporating fermented finger millet flour or sourdough into wheat-based dough.	Ragi sourdough bread, Multigrain bread	Improved texture, taste, and shelf life; higher antioxidants (57).
Fermented pancakes/flatbreads	Made by mixing finger millet flour with urad dal and allowing it to ferment before cooking.	Ragi dosa, Ragi idli.	Fluffy texture, enhanced digestibility, rich in B-vitamins (57).
Fermented finger millet yogurt (dairy-free).	Plant-based yogurt alternative prepared by inoculating finger millet slurry with probiotics.	Ragi probiotic yogurt (31).	Suitable for vegans, good for lactose-intolerant individuals (31, 61).
Fermented snacks (crackers, crisps).	Finger millet fermented dough used to prepare crunchy snack products.	Ragi khakhra, fermented millet crisps.	Improved flavor, texture, and nutritional quality (61).
Finger millet-based fermented composite flour.	Fermented finger millet flour blended with other cereals and legumes for complementary feeding or baking.	Multigrain weaning mix, infant cereal.	Balanced amino acids, high calcium, protein rich (15, 61).

### 2.4 Soybean

**Figure 3:**
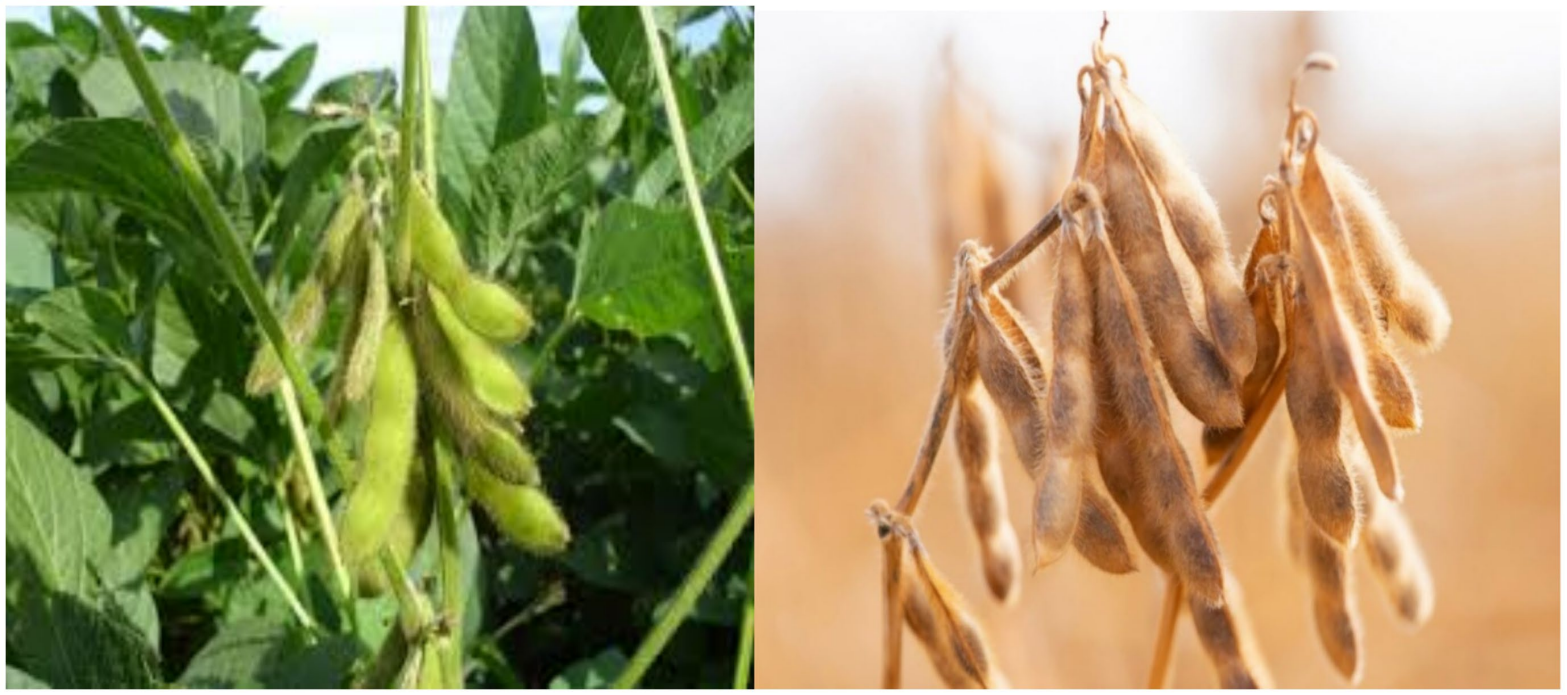
Colour change in soybean pods and seeds from green to yellowish-brown at maturity.

Soybean is one of the most important leguminous crops in South Africa because of its high protein content, making it essential for poultry feed. Rising demand for poultry products has fuelled swift growth in the soybean industry, making it a cornerstone of domestic animal feed production. Figure 3 illustrates soybean mature in the field; both the pods and seeds exhibit a notable-color change, shifting from green to yellowish brown (18). The South African poultry industry is the largest segment of the country’s agricultural sector and is essential to national food and nutrition security, since poultry meat comprises about 60% of total per capita meat consumption. Therefore, supporting this industry is essential to the nation’s well-being (18). Given this high consumption rate, soybean has emerged as a strategic crop. However, South Africa’s soybean production, currently at approximately 1.96 million tonnes annually, falls short of meeting poultry industry’s growing demand (19). The total soybean demand for the 2023/24 marketing season is projected at 2.31 million tonnes, revealing a substantial supply gap (19). Therefore, immediate action is needed to address this shortfall and ensure market stability (19).

#### 2.4.1 Origin and distribution of soybean

Soybean, a high-value crop in the Fabaceae family, originated in northern China, where it was domesticated over 4,000 years ago and became a cornerstone of agriculture, nutrition, and trade in ancient civilizations (20). From its origin, it gradually spread across East and Southeast Asia before being introduced to the Americas, where countries such as the United States, Brazil, and Argentina have become major producers. However, it is recognized as one of the most economically and nutritionally significant crop worldwide (20). Its global importance is largely attributed to its exceptional protein content, especially at a time when the cost of traditional protein sources like meat, dairy, fish, and eggs continues to rise (20).

#### 2.4.2 Taxonomic and botanical description of soybean

*Glycine max* is a crop of major significance among economically and nutritionally important legumes (21). It is diploid species, with 2n = 40 chromosomes. Soybeans, as legumes, have the unique capacity to fix atmospheric nitrogen via symbiosis with bacteria, hence increasing soil fertility, Table 6 shows the taxonomic classification of soybean (21). *Glycine max* is classified as a member of the kingdom Plantae, subkingdom Tracheobionta (vascular plants), superdivision Spermatophyta (seed plants), division Magnoliophyta (angiosperms), class Magnoliopsida (dicots), subclass Rosidae, order Fabales. Family Fabaceae, subfamily Faboideae, tribe Phaseoleae, genus *Glycerine*, and subgenus Soja.

**Table 6:** Biological activities, health benefits and significance of phytochemicals in soybean.

Secondary metabolite	Chemical classification	Biological role in soybean	Health benefits in humans	Contribution to soybean’s nutritional and agricultural value
Isoflavones (e.g., genistein, daidzein, glycitein).	Polyphenolic phytoestrogens.	Protection from UV radiation, pathogen defence.	Antioxidant, phytoestrogenic activity, cancer prevention, cardiovascular health (22).	Enhance marketability due to their therapeutic use in functional foods; increase consumer demand.
Tocopherols (Vitamin E compounds).	Lipid-soluble antioxidants.	Membrane protection against oxidative stress.	Antioxidant, slows aging, supports immune function.	Adds value for health-conscious markets; contributes to oil stability and shelf life.
Phenolic acids (e.g., caffeic acid and ferulic acid).	Aromatic phenols	Antimicrobial defense, UV protection.	Antioxidant, anti-inflammatory, protect against metabolic syndromes (22).	Improve functional profile of processed/fermented soybean products.
Anthocyanins (in black soybeans)	Water-soluble flavonoids.	Pigmentation and stress resistance.	Anti-inflammatory, vision support, neuroprotective.	Drive niche markets for specialty soy varieties; increase biodiversity in cultivation.
Tannins	Polyphenolic macromolecules.	Defense against herbivores and pathogens.	Antioxidant, antimicrobial, anti-carcinogenic effects.	May influence feed and food formulations; present challenges for digestibility if not processed.
Vitamins (e.g., Vitamin C, B-complex).	Water-soluble micronutrients.	Metabolic co-factors, stress resilience.	Immune support, nervous system function, energy metabolism.	Boost soybean’s profile as a nutrient-dense legume, aiding food security.

Adapted from Verma et al. (9); Messina (22).

#### 2.4.3 Secondary metabolites of soybean

Soybean harbors a range of bioactive compounds explained in Table 7, such as isoflavones, tocopherols, phenols, anthocyanins, tannins, and various vitamins, contributing to its antioxidant capacity and overall health benefits soybean’s farming and nutritional demand has led to extensive cultivation and consumption (9, 22).

**Table 7:** Secondary metabolites in soybean.

Secondary metabolite class	Specific compounds/metabolites	Reported range/levels	Biological/functional role
Isoflavones	Daidzein, Genistein, Glycitein, Coumestrol.	Daidzein: (1.2–8.9 mg/g DW). Coumestrol: (0.3–4.8 mg/g DW). Total isoflavones typically (0.8–3.3 mg/g DW).	Phytoestrogenic; antioxidant; anti-cancer; cardiovascular benefits (58, 59)
Phenolic acids	Ferulic acid, p-coumaric acid, caffeic acid, gallic acid, chlorogenic acid.	Total phenolic content: (1.15–1.77 mg GAE/g)	Antioxidant; anti-inflammatory; antimicrobial (58, 59)
Flavonoids	Quercetin, Kaempferol, Catechin, Apigenin, Luteolin.	Flavonoid content: (0.68–2.13 mg QE/g)	Antioxidant; anti-inflammatory; neuroprotection (58, 59)
Tannins	Condensed and hydrolyzable tannins.	Detected, variable among genotypes	Antimicrobial; antioxidant; potential anti-nutritional factors (58, 59)
Phytosterols	B-Sitosterol, Campesterol, Stigmasterol.	Typically (0.20–0.45%) of seed dry weight	Cholesterol lowering; anti-cancer; cardiovascular support (58, 59)
Saponins	Soyasaponins (group A, B, E, DDMP).	(0.1–0.8%) of dry weight.	Cholesterol reduction; immunomodulatory; anti-cancer (58, 59)
Alkaloids	Canavanine, other uncharacterized alkaloids.	Trace to low levels; qualitative identification.	Antimicrobial; defensive; potential toxicity.
Terpenes/Terpenoids	Monoterpenes, sesquiterpenes, triterpenes, phytol.	Detected, variable (qualitative and quantitative).	Aromatic, antioxidant, defense-related effects (59).
Coumarins	Coumarin, umbelliferone, scopoletin.	Detected; range variable.	Anticoagulant; antioxidant; defense (59).
Peptides	Lunasin, Bowman-Birk inhibitor.	Peptide fractions vary by genotype and process.	Anticancer; anti-inflammatory; antioxidant (58).
Others (minor classes)	Organic acids, aldehydes, ketones, esters, heterocyclic and amide compounds.	Many identified (88+ by GC-MS across genotypes).	Flavor, antimicrobial, antioxidant, metabolic functions (58, 59)

Nevertheless, its nutritional value is partially restricted by the presence of naturally occurring antinutrients, including phytates, oxalates, saponins, tannins, gums, and trypsin inhibitors; consequently, these compounds can impede mineral absorption and reduce protein digestibility (9, 10). To overcome these limitations and simultaneously enhance key traits such as yield, pest resistance, and functional quality, genetic modification has been employed to develop a range of superior crop varieties (23). These are used in the production of diverse food products including soymilk, tofu, flour, oils, and fortified cereals. The nutritional and phytochemical properties of these varieties are also shaped by environmental and climatic conditions, adding further complexity to their composition and functionality (23). Table 7 presents a detailed overview of secondary metabolites in soybean.

#### 2.4.4 Nutritional constituents of soybean

Soybean is a crop that delivers all essential amin acids, classifying it as a complete protein, though it is relatively limited in sulphur-containing amino acids like methionine and cysteine (24). Moreover, proteins account for approximately 37%–42% of its dry weight, primarily comprising the storage globulins 11S glycinin and 7S *β*-conglycinin, which are fundamental to its nutritional and functional value (24). In addition, the crop contains major proteins such as protease inhibitors that influence enzymatic activity (8). In addition to its high protein content, soybean contains about 20% unsaturated fats (Table 8), 30% carbohydrates, 17% dietary fiber—both soluble and insoluble—and 5% ash, as indicated in Table 8 (24). This composition makes it a potent source of essential vitamins and minerals including calcium, magnesium, potassium, iron, and zinc as shown in Table 8 (8, 24).

**Table 8:** Functional role of soybean nutritional constituents.

	Functional role
Fatty acids*	
Linoleic acid (ω-6)	Essential fatty acid
Oleic acid (ω-9)	Heart-healthy fat
Linolenic acid (ω-3)	Anti-inflammatory
Fibre**	
Soluble fibre	Lowers blood cholesterol
Insoluble fibre	Enhances bowel movement
Minerals**	
Calcium	Bone and muscle function
Magnesium	Enzyme cofactor, heart rhythm
Potassium	Fluid balance, nerve transmission
Iron	Haemoglobin synthesis
Zinc	Immune function

Source: **Lee and Kim (8), *Aanchal et al. (24).

As shown in this table, the favorable omega-6 to omega-3 ratio makes soy oil a healthy dietary fat (8). In addition, soybean provide ~30% carbohydrates, such as (a) stachyose and raffinose: oligosaccharides that may cause flatulence but act as prebiotics. (b) Dietary fibre: Constitutes about 17% of the seed mass (24). Also, soybean is rich in both macronutrients and micronutrients, vital for metabolic and enzymatic functions (24).

### 2.5 Soybean fermented products

The traditional and modern fermented soybean products are produced and consumed all over the world, but mainly in East and Southeast Asian countries such as Korea, China, Japan, Indonesia, and Vietnam (25). The most common soybean fermented foods are soy sauce, miso, tempeh, natto, fermented tofu (furu), doenjang, kinema, cheonggukjang, sufu, and tofuyo (Table 9) (25–27).

**Table 9:** An overview of traditional fermented soybean products: processes, nutritional benefits, and functional properties.

Product	Overview	Fermentation process	Nutritional/functional aspects
Soy sauce	A staple Asian condiment made by fermenting soybeans and wheat.	Two-stage process: Koji fermentation using *Aspergillus oryzae/sojae*, followed by brine fermentation with *Zygosaccharomyces rouxii*, LAB, and yeasts.	Rich in amino acids, peptides, and antioxidants. Exhibits anti-inflammatory and anti-hypertensive effects (25).
Miso	Japanese soybean paste used in soups and sauces.	*A. oryzae* for koji fermentation, followed by LAB (*Tetragenococcus halophilus*).	High in protein, vitamins, probiotics; supports gut health and reduces cancer risk (25, 26).
Tempeh	Indonesian product with nutty flavor and firm texture.	Solid-state fermentation with *Rhizopus oligosporus*.	Enhanced protein digestibility, reduced anti-nutrients, increased folate and B12 (26, 27).
Natto	Sticky Japanese food with strong flavor.	Fermentation by *Bacillus subtilis natto*.	Rich in vitamin K2 and nattokinase (supports cardiovascular health) (27).
Fermented Tofu (Furu)	Also called Chinese cheese, soft and creamy.	Mold fermentation (*Actinomucor elegans*, *Mucor* spp.) followed by brining with wine and spices.	Contains peptides, amino acids, antioxidants; exhibits antimicrobial potential (25, 26).
Doenjang	Korean paste used in stews and side dishes.	Fermented meju blocks with *Bacillus*, *Aspergillus*, and LAB.	Rich in isoflavones, saponins; has anticancer and anti-inflammatory effects (25, 26).
Kinema	Nepali fermented soy with sticky texture.	Natural fermentation by *Bacillus subtilis*.	Important protein source in Himalayan diets, rich in beneficial microbes (27).
Cheonggukjang	Korean quick-fermented soybean product.	Short fermentation (2–3 days) with *Bacillus subtilis*.	Contains polyglutamic acid and nattokinase; promotes digestion and cardiovascular health (26).
Sufu	Chinese fermented tofu with pungent aroma.	Like Furu: mold fermentation, then aging in brine.	Probiotic, antioxidant, and proteolytic activity (25).
Tofuyo	Okinawan fermented tofu aged in rice wine.	Mold fermentation followed by aging with red yeast (*Monascus* spp.) and alcohol.	Rich in peptides and functional enzymes; supports liver and gut health (25, 27).

### 2.6 Nutritional impacts

Finger millet and soybean serve as core ingredients in a wide range of dairy-free fermented foods, including yoghurt alternatives, kefir-like beverages, and tempeh-based spreads. Notably, their combination enhances both nutritional quality and functional properties, making them especially valuable for developing innovative, plant-based products that meet diverse dietary needs (28, 29). They are especially beneficial for lactose-intolerant people who require nutritious non-dairy alternatives. Finger millet is naturally gluten-free, high in fiber, calcium, iron, and polyphenols (28). Fermentation increases nutrient bioavailability, and enhances prebiotic activity (7, 30). Table 9 provides a detailed explanation of these benefits (7, 30). Soybean’s blending and gel-forming properties improve creaminess, structure, probiotic viability, shelf life, and sensory qualities, supporting fermentation’s role in sustainable food systems (6, 8). Scientifically, finger millet increases viscosity and water holding, increasing the texture of both spoonable and drinkable products (7). Soybean’s superior blending and gel-forming properties enhance creaminess, structure, probiotic viability, shelf life, and sensory qualities, aligning with fermentation’s critical role in sustainable food systems (6, 8). While adhering to environmental and health benefits, it contributes to the development of different nutrient-dense food products and presents encouraging responses to the impending protein crisis (6).

However, in this review, it is important to note that many reported health benefits associated with fermented finger millet and soybean products are derived from *in vitro* or small-scale experimental studies (Table 10). Direct clinical evidence in human populations remains limited. Therefore, claims regarding probiotic effects, chronic disease prevention, or metabolic benefits should be interpreted as promising but not yet conclusively established. Therefore, this creates a critical gap to be filled by future studies.

**Table 10:** Comparative benefits of finger millet and soybean in fermented foods.

Aspect	Finger millet	Soybean	Combined benefit in fermented food
Nutritional profile	High in calcium, iron, fiber, polyphenols; gluten-free (30).	High in protein; contains bioactive peptides and aglycone isoflavones (24).	Provides essential nutrients for lactose-intolerant individuals (6).
Effect of fermentation	Improves mineral bioavailability, lowers antinutrients, enhances prebiotic value (30).	Enhances digestibility; increases bioactive compounds (8).	Supports gut health and nutrient absorption (6).
Textural contribution	Increases viscosity and water retention.	Offers creaminess, emulsifying, and gel-forming properties.	Enhances mouthfeel, texture, and consistency of spoonable/drinkable products (7).
Product applications	Yogurt alternatives, kefir-like beverages, fermented porridge (28, 29)	Yogurt alternatives, tempeh-based spreads, kefir-like beverages (29).	Diverse dairy-free fermented food option.
Probiotic & shelf-life support	Supports probiotic growth and stability.	Stabilizes structure and improves shelf life (6).	Increases product viability and sensory appeal.
Sustainability contribution	Naturally resilient crop; supports food security.	Addresses protein crisis; sustainable plant protein source (30).	Contributes to sustainable, health-aligned food systems.

Aanchal et al. (24); Abbaspour (28); Devi et al. (7); Ganzle et al. (30); Lee and Kim (8); Patel et al. (6).

### 2.7 Microbial mechanisms

In plant-based foods made from finger millet and soybean, lactic acid fermentation significantly improves digestibility, reduces antinutritional factors, and increases the bioavailability of essential nutrients (28). These modifications benefit individuals with lactose intolerance and plant-based diets by enhancing nutrient absorption and promoting gastrointestinal health (28). In addition to its nutritional benefits, lactic acid fermentation enhances the functional and sensory qualities of plant-based products by improving taste, texture, and aroma (29). This method aligns with the growing demand for functional foods that cater to health-conscious consumers. The environmental sustainability of finger millet and soybean, due to their low resource demands, supports their inclusion in sustainable food systems, while both lactic acid and acetic acid fermentation further enhance the functional qualities of plant-based foods (28, 29, 31). This transformation not only imparts a characteristic tangy flavor but also acts as a natural preservative, thereby extending the shelf life of fermented products (31). When integrated with lactic acid fermentation in the production of plant-based foods, acetic acid fermentation contributes to a more complex flavor profile and improved microbial stability (31). The synergistic application of both lactic and acetic acid fermentation in finger millet and soybean-based foods presents a comprehensive and sustainable approach to food processing (28). This dual fermentation strategy not only enhances the nutritional and sensory properties of plant-based products but also increases their market appeal and shelf stability. As a result, these innovations meet the needs of diverse consumers, including those seeking lactose-free, plant-based options, while also supporting environmentally sustainable food production (28).

### 2.8 Product categories

Despite the growing interest in using finger millet and soybean as functional ingredients in fermented plant-based foods, several implementation challenges prevent mainstream adoption and commercialization (28). One of the major concerns is the standardization of the fermentation process. However, a significant concern is the existence of anti-nutritional components, including phytic acid, tannins, and enzyme inhibitors, in both finger millet and soybean. These compounds can reduce nutritional bioavailability and protein digestibility (32). Furthermore, finger millet contains a high dietary fiber content and is low in gluten, which can impact the texture and structure of the final fermented products (7). Soybean can contribute beany flavors that are undesirable to some people (7). Given these sensory limitations, advanced processing strategies such as mixed cultures, enzymatic treatments, and flavor-masking techniques, are required to enhance product acceptability (7). There are infrastructure and economic constraints, particularly in low-and middle-income countries where these crops are most common (7). Limited access to fermentation technologies, cold chain systems, and quality control procedures can limit the scalability and market reach of novel fermented products (33). Addressing these implementation problems is critical to fully utilizing the nutritional and physiological potential of finger millet and soybean in the creation of next-generation fermented foods (33). From a translational perspective, the findings reviewed here have strong relevance for community level small scale food processing. Fermentation of finger millet and soybean can be implemented using low-cost starter cultures and locally available raw materials. However, barriers including limited infrastructure and quality control systems remain constrains that must be addressed to enable commercial uptake.

### 2.9 Processing challenges

Finger millet and soybean deliver outstanding nutrition, proving high levels of protein, fiber, essential amino acids, and critical micronutrients like calcium, iron, and zinc (Devi et al., 202). Finger millet and soybean contain antinutritional factors—including phytic acid, tannins, oxalates, and enzyme inhibitors, as shown in Table 11—that hinder mineral absorption and protein digestibility (34, 35). To maximize the nutritional value of finger millet and soybean, it is crucial to their antinutrients (35). However, there are many techniques for removing antinutritional elements, including soaking, fermentation, germination, roasting, and enzymatic treatment, which are all beneficial to the process. This makes plant-based foods easier to digest, makes minerals more available, and improves their overall nutritional quality (34, 35).

**Table 11:** Comparison of common antinutrients in finger millet and soybean.

Antinutrient	Presence in finger millet	Presence in soybean	Nutritional impact	Reduction methods
Phytates	High	High	Binds minerals like iron, zinc, and calcium, reducing their bioavailability.	Soaking, germination, fermentation, enzymatic treatment (34, 35).
Tannins	High	Low	Inhibits protein digestibility and iron absorption.	Dehulling, soaking, fermentation, cooking (34).
Oxalates	Moderate	Low	Interferes with calcium absorption, can contribute to kidney stone formation.	Boiling, soaking, fermentation (34, 35)
Trypsin inhibitors	Absent/Low	High	Inhibits protein-digesting enzyme trypsin, reducing protein utilization	Heat treatment, fermentation, germination (34, 35).
Lectins	Absent	Moderate	Can cause digestive discomfort and interfere with nutrient absorption	Proper cooking, soaking, fermentation (34, 35)
Saponins	Low	High	Bitter taste, can interfere with nutrient absorption at high levels	Washing, fermentation, heat treatment (34, 35).

## 3 Knowledge gaps in current science and technology

### 3.1 Microbial ecology of fermentation

Although finger millet and soybean fermentations are characterized by diverse and complex microbial communities, comprehensive characterization of these communities remains incomplete (36). *Lactobacillus*, *Pediococcus*, and *Leuconostoc* are among the most common genera found in fermented foods. However, the specific potential roles and interactions of these microorganisms in finger millet and soybean fermentations are not established (36). Advanced metagenomic and metabolomic methods are required to understand the functional dynamics of these microbial consortia (36, 37).

Recent advances in high throughput sequencing and multi-omics technologies have significantly deepened understanding of microbial community dynamics during fermentation. Metagenomic analysis, in particular, have been used to characterize complex consortia of bacteria and fungi that drive biochemical transformations in plant-based substrates. For example, shotgun metagenomics has revealed that only taxonomic profiles but also functional potential of microbiomes in cereal and legume fermentations, enabling identification of genes linked to carbohydrate metabolism, proteolysis, and anti-nutrient degradation (38, 39). These studies demonstrate that fermentation is governed by dynamic succession of microbial taxa, with keystone species shifting as substrate composition and environmental conditions change. Moreover, meta-transcriptomic and metabolomic profiling have revealed active metabolic pathways and correlated metabolite outputs, underscoring the value of integrated omics for linking community structure with functional outcomes (40). Incorporating these approaches addresses gaps in traditional culture-dependant methods, which often underestimate the diversity and activities of non-cultivatable microorganisms.

### 3.2 Limited characterization of bioactive metabolites

Fermentation improves the nutritional value by increasing the availability of bioactive compounds (41). Specifically, fermentation not only enhances phenolic compounds, flavonoids, and tocopherols in soy-based beverages (41), but also significantly increases total polyphenolic and flavonoid content in fermented finger millet flours. Despite these discoveries, there has been a lack of extensive research on the specific bioactive metabolites created during fermentation and their health implications (37). Partial hydrolysis of protein and carbohydrates produces intermediate peptides and oligosaccharides whose physiological effects remain uncertain (42). Advanced metabolomic approaches are required to fully characterise these pathways and confirm the extent of nutrient transformation.

### 3.3 Underexplored socioeconomic and cultural dynamics

Consumer acceptance of fermented products from finger millet and soybean is strongly influenced by cultural norms, socioeconomic factors, and public awareness of their health benefits (43). Although these products offer notable nutritional advantages, their integration into markets where they are not traditionally consumed may encounter challenges due to unfamiliarity and potential cultural resistance (37). To facilitate broader adoption and successful commercialization, it is essential to undertake in-depth investigations into consumer behavior, market trends, and cultural attitudes (37). Therefore, when examining consumer responses to novel food products, it is crucial to account for variables such as age, gender, and regional dietary habits, as these elements significantly shape purchasing behavior and perceptions, thereby providing valuable insights for developing effective marketing approaches and culturally relevant product innovations (37). Furthermore, educational campaigns and community engagement programs can be instrumental in demystifying fermented foods and promoting their health benefits, thereby fostering positive consumer attitudes, and increasing market penetration (37). Collaborative efforts involving local influencers, healthcare professionals, and food producers may also enhance trust and drive acceptance across diverse demographic segments (37).

### 3.4 Methodological standardization needs in fermented food research

Future research on the fermentation of plant-based foods such a finger millet and soybean require far greater methodological harmonization to enhance comparability, reproducibility, and translational relevance. At present, significant variability exists in experimental designs, fermentation conditions, analytical techniques, and reporting standards, which limits the ability to synthesize results across studies. For example, different studies rely on a wide range of sample collection methods, fermentation times, starter culture selections, and analytical assays, making it difficult to compare outcomes regarding nutrient bioavailability and microbial activity, for example, differences in analytical protocols for assessing anti nutrients and functional compounds. Standardized fermentation protocols are needed with respect to substrate preparation, inoculum sourcing, starter culture characterization, fermentation time and temperature post-processing conditions. The absence of uniform procedures often results in highly divergent outcomes even when similar raw materials are used, which restricts evidence synthesis and reduces confidence in conclusions drawn across different laboratories.

Equally important is the need for harmonized frameworks. Recent revises of plant based fermented food emphasize that current methods for measuring physicochemical properties, anti-nutritional factors, sensory characteristics, and functional metabolites often lack consistent calibration standards such as chromatographic and spectrometric techniques (44). This diversity in analytical approaches complicates cross-study comparison and meta interpretation of data. Harmonization of analytical endpoints such as protocols for mineral bioavailability assays, protein digestibility measurements, and metabolite profiling would enable more robust meta-analysis and facilitate clearer interpretation of functional outcomes (45). In particular, adoption of internationally recognized analytical standard and cross laboratory validation studies should be prioritized to strengthen data quality and comparability.

Moreover, standardized reporting formats are essential to improve transparency and reproducibility. Many studies provide incomplete descriptions of critical methodological details such as microbial strain genotypes, inoculum densities, fermentation parameters, and statistical analysis methods, which impedes replication. Development of minimum reporting guidelines, like CONSORT, PRISMA, or other discipline specific standards would improve clarity around methodological choices, enhance reproducibility, and support systematic evidence synthesis in the field (46). Clearly defined outcome e measures, consentient units of reporting, and comprehensive metadata documentation should be incorporated into future publications to support data reuse and integration across research programs.

Addressing these methodological gaps is essential for advancing fermented food research from descriptive and exploratory studies toward more rigorous, comparative, and scalable science. Without harmonized methodologies and standardized reporting, the ability to translate promising laboratory findings into evidence based applications for nutrition, health, and industry will remain constrained.

## 4 Future directions for research, innovation, and policy

### 4.1 Functional genomics and mechanistic insights

There are currently no commercially established fermented products for finger millet and soybean, despite their exceptional nutritional potential. Established success with lactic and propionic acid bacteria in pulse-based fermentations underscores the effectiveness of microbial optimization (36). It is crucial to create specialized microbial consortia for finger millet and soybean. This will assure consistent quality, increased nutritional content, and higher cultural acceptance of underutilized crops in fermented foods markets (36).

Beyond taxonomic compositions, omics-based studies have unlocked mechanistic insights into how specific microbial taxa contribute to fermentation traits. Functional genomics, including metagenome assembled genomics (MAGs) and gene expression profiling, have been applied to identify enzymes responsible for starch hydrolysis, proteases activity, and antinutrient breakdown in complex fermentations (47, 48). Proteomics analyses further elucidate the expression of key metabolic proteins under different fermentation conditions, offering a systems level view of microbial contributions to flavor development and nutritional enhancement. Integrative metabolomics has likewise mapped biochemical changes, linking specific metabolites with microbial activity and host sensory outcomes. Such omics platforms are transforming the ability to pinpoint casual relationships between microbial functions and product phenotypes, which traditional microbiological assays alone cannot solve.

### 4.2 Optimizing processes using emerging technologies

The innovative processing technologies, such as enzymatic pre-treatments and precision fermentation (explained in detailed Sections 4.3 and 4.4), can provide opportunities to improve the nutritional and sensory properties of fermented foods (49). These techniques reduce antinutrients while increasing nutrient absorption, improving both the nutrition and flavor of fermented finger millet and soybean (49).

### 4.3 Enzymatic pre-treatment

Enzymes such as phytase, cellulase, and protease are required to break down antinutritional substances such phytates, tannins, and non-starch polysaccharides, which impede mineral absorption and protein digestion (49). Phytase increases iron and zinc bioavailability by releasing bound phosphorus, whereas protease increases amino acid availability while also improving the texture and flavor of soybean products (49). Optimizing these enzymic treatments by manipulating pH, temperature, and reaction time can dramatically improve substrate quality prior to fermentation (49).

### 4.4 Precision fermentation

According to Jahn et al. (49), the accuracy of fermentation provides more control and customization. However, it also involves selecting and engineering specific strains of beneficial microbes, such as *Lactobacillus plantarum*, and *Bacillus subtilis*, or *Propionibacterium freudenreichii,* to consistently produce desired compounds like bioactive peptides, vitamin-B, (GABA) y-aminobutyric acid (49). These metabolites improve both nutritional value and desirable sensory attributes such as umami flavor and creamy texture, while allowing for real-time monitoring and control of fermentation to ensure consistent, and high-quality products (49). It will enable the development of consistent, functional, and culturally relevant fermented products with enhanced health benefits, greater market appeal, and commercial scalability.

### 4.5 Innovation in product design and diversification

The innovation in product design modifies the form, content, and delivery of food to satisfy changing consumer needs for convenience, health, and sensory appeal (49). Diversification is the expansion of the spectrum of food products by various component combinations, fermentation processes, functional aims, and cultural adaptability (49). These techniques are critical for improving marketability, accessibility, and functional value. Key drivers of innovation include the growing demand for plant-based, gut-friendly foods, enhanced nutritional security, and sustainable use of local crops. Although there are still issues with microbial viability, consumer acceptance, standardizing the fermentation process, and preserving both nutritional value and sensory quality (49).

### 4.6 Strengthening the sustainability and food sovereignty agenda

Utilizing locally grown finger millet and soybeans in fermented foods supports sustainable agriculture and food sovereignty, while their climate resilience also strengthens food security in regions like Sub-Saharan Africa (1, 50). Promoting their use in functional foods can improve nutritional outcomes and enhance local economies (50). Finger millet’s drought tolerance in the Limpopo province of South Africa supports sustainable agriculture and nutritional security. Its resilience benefits smallholder farmers, while intercropping increases yields and improves soil fertility (51–53). Similarly, soybean trials are being done throughout South Africa’s several provinces, including Mpumalanga, KwaZulu-Natal, and the Eastern Cape, through both government and commercial sector initiatives (51, 53). These trials seek to assess enhanced cultivars that are not just high-yielding but also disease-resistant and suited for low-input farming systems (52). Soybeans in crop rotation systems have showed potential for enhancing soil nitrogen levels through biological nitrogen fixation, lowering the demand for synthetic fertilizers, and promoting sustainable soil management techniques (53).

### 4.7 Policy advocacy and multi-stakeholder engagement

The South African government promotes indigenous crops with varied value chains to increase food security and assist rural populations (54). Policies outlined in the Natural Development Plan (NDP) and the Agricultural Policy Action Plan (APAP) prioritize support for smallholder farmers, including growth, and the development of agroprocessing opportunities (54). However, the Department of Agriculture, Land Reform and Rural Development (54) encourages multistakeholder collaboration through initiatives such as the Agricultural Sector Master Plan, which brings together government, industry, academia, and civil society to define sector priorities (54). Against this backdrop, the synergistic effect of policy and stakeholder involvement improves the economic viability and consumer acceptability of functional foods (49). Furthermore, developing geographical indication (GI) tags for indigenous finger millet varieties, encouraging contract for soybean, and assisting local food small and medium-sized enterprises (SMEs) with capacity building can all increase their competitiveness in domestic and foreign markets, facilitate product tracking, and foster trust (36). However, the product inclusive policy conversations with local communities, traditional knowledge holders, and gender-focused groups ensure that innovation processes are equitable and culturally appropriate, particularly in areas where finger millet is a common food (49).

## 5 Conclusion and prospects

Integrating finger millet and soybean into fermented food systems provides a scientifically robust solution to food and nutrition security by leveraging their complementary nutrient profiles and adaptability to challenging agroecosystems. Fermentation further enhances digestibility and nutrient bioavailability, making this approach relevant for addressing malnutrition, climate resilience, and the demand for functional, plant-based foods across diverse cultures. Through the lens of food biotechnology, this study has highlighted how the synergistic effects of fermentation enhance the nutritional and functional properties of these crops. However, this advancement is especially relevant for tackling lactose intolerance and micronutrient deficiencies. The emphasis on locally adapted crops and traditional techniques, combined with innovative processing methods that supports the development of sustainable and health-promoting food systems that can cater to both urban and rural populations. Integrating finger millet and soybean into fermented foods presents strong potential for both nutritional improvement and innovative product development. Research should now focus on optimizing fermentation parameters and starter cultures specific to these substrates to maximize nutritional yields and sensory acceptance. Additionally, scaling up the development of these fermented products through inclusive value chains can empower smallholder farmers and promote regional food sovereignty. Moreover, integrating these crops into climate-smart practices like intercropping, agroforestry, and diversified farming enhances both ecological resilience and food diversity. From a public health perspective, advancing awareness and accessibility of such nutritionally enhanced fermented foods could play a pivotal role in non-communicable disease prevention and dietary improvement strategies across vulnerable communities. Ultimately, integrating finger millet and soybean in fermented foods can catalyze a shift toward more sustainable, equitable, and health-driven food production.

## Data Availability

The original contributions presented in the study are included in the article/supplementary material, further inquiries can be directed to the corresponding author/s.
